# PhIP-Seq Reveals Autoantibodies for Ubiquitously Expressed Antigens in Viral Myocarditis

**DOI:** 10.3390/biology11071055

**Published:** 2022-07-13

**Authors:** Mahima T. Rasquinha, Ninaad Lasrado, Erika Petro-Turnquist, Eric Weaver, Thiagarajan Venkataraman, Daniel Anderson, Uri Laserson, H. Benjamin Larman, Jay Reddy

**Affiliations:** 1 School of Veterinary Medicine and Biomedical Sciences, University of Nebraska-Lincoln, Lincoln, NE 68583, USA; mrasquinha2@huskers.unl.edu (M.T.R.); ninaad@huskers.unl.edu (N.L.); 2 Center for Virology and Vaccine Research, Beth Israel Deaconess Medical Center, Harvard Medical School, Boston, MA 02215, USA; 3 Nebraska Center for Virology, University of Nebraska-Lincoln, Lincoln, NE 68583, USA; epetro-turnquist2@huskers.unl.edu (E.P.-T.); eweaver2@unl.edu (E.W.); 4 Division of Immunology, Department of Pathology, Johns Hopkins School of Medicine, Baltimore, MD 21205, USA; tvenkat2@jhmi.edu; 5 Department of Internal Medicine, Division of Cardiovascular Medicine, University of Nebraska Medical Center, Omaha, NE 68198, USA; danderso@unmc.edu; 6 Department of Genetics and Genomic Sciences and Precision Immunology Institute, Icahn School of Medicine at Mount Sinai, New York, NY 10029, USA; uri@lasersonlab.org

**Keywords:** coxsackievirus B3, myocarditis, viral myocarditis, PhIP-Seq, autoantibodies, autoimmune myocarditis, animal models of myocarditis

## Abstract

**Simple Summary:**

Myocarditis is the inflammation of the heart muscle, and viral infections are a common cause of this disease. Myocarditis in some patients can progress to dilated cardiomyopathy (DCM). The mouse model of coxsackievirus B3 (CVB3) is commonly used to understand this disease progression in DCM patients. In this paper, we have attempted to analyze antibodies for heart antigens that could be produced as a result of heart damage in animals infected with CVB3 using a technique called Phage ImmunoPrecipitation Sequencing (PhIP-Seq). The analyses led us to identify antibodies for several proteins that were not previously reported that may have relevance to human disease.

**Abstract:**

Enteroviruses such as group B coxsackieviruses (CVB) are commonly suspected as causes of myocarditis that can lead to dilated cardiomyopathy (DCM), and the mouse model of CVB3 myocarditis is routinely used to understand DCM pathogenesis. Mechanistically, autoimmunity is suspected due to the presence of autoantibodies for select antigens. However, their role continues to be enigmatic, which also raises the question of whether the breadth of autoantibodies is sufficiently characterized. Here, we attempted to comprehensively analyze the autoantibody repertoire using Phage ImmunoPrecipitation Sequencing (PhIP-Seq), a versatile and high-throughput platform, in the mouse model of CVB3 myocarditis. First, PhIP-Seq analysis using the VirScan library revealed antibody reactivity only to CVB3 in the infected group but not in controls, thus validating the technique in this model. Second, using the mouse peptide library, we detected autoantibodies to 32 peptides from 25 proteins in infected animals that are ubiquitously expressed and have not been previously reported. Third, by using ELISA as a secondary assay, we confirmed antibody reactivity in sera from CVB3-infected animals to cytochrome c oxidase assembly factor 4 homolog (COA4) and phosphoinositide-3-kinase adaptor protein 1 (PIK3AP1), indicating the specificity of antibody detection by PhIP-Seq technology. Fourth, we noted similar antibody reactivity patterns in CVB3 and CVB4 infections, suggesting that the COA4- and PIK3AP1-reactive antibodies could be common to multiple CVB infections. The specificity of the autoantibodies was affirmed with influenza-infected animals that showed no reactivity to any of the antigens tested. Taken together, our data suggest that the autoantibodies identified by PhIP-Seq may have relevance to CVB pathogenesis, with a possibility that similar reactivity could be expected in human DCM patients.

## 1. Introduction

Myocarditis is an inflammation of the heart muscle that can lead to dilated cardiomyopathy (DCM) [1,2,3]. Various infectious and non-infectious causes have been implicated in the development of myocarditis, with viruses being the commonly suspected agents of the disease [1,2,3,4]. Historically, enteroviruses and adenoviruses were believed to be the key triggers of myocarditis in North America and Europe, respectively [5,6], but this trend is changing as other viruses, such as human herpesvirus-6 and parvovirus-19, are increasingly being reported as triggers, with the recent addition to the list being severe acute respiratory syndrome coronavirus-2 [7,8,9]. Mechanistically, different viruses cause tissue destruction by different pathways, but all appear to have a common outcome: DCM. This notion is supported by the fact that infectious viruses are rarely detected in affected patients, and disease associations are generally made based on signatures such as virus-reactive antibodies and viral nucleic acids [6,10]. Thus, determination of a cause-and-effect relationship is difficult to establish in organ-specific diseases such as myocarditis.

To understand the pathogenesis of viral myocarditis, the mouse model of coxsackievirus B (CVB)3 infection is commonly employed. The disease course in susceptible strains (A/J and BALB/c mice) assumes acute (or viral) and chronic (or non-viral) phases, occurring along the continuum [1,11,12], whereas C57Bl/6 mice are relatively resistant, developing only acute myocarditis [13]. Since the histological features of post-infectious myocarditis induced with CVB3 in susceptible mice resemble those seen in human disease, this mouse model is helpful to delineate the pathogenetic events in viral myocarditis [11].

Mechanistically, autoimmunity has been proposed as a mechanism for the development of post-viral myocarditis, as demonstrated by the presence of antibodies for select antigens, such as cardiac myosin (Myhc), adenine nucleotide translocator (ANT), branched-chain α-ketoacid dehydrogenase kinase (BCKDk) [10], Sarcoplasmic/endoplasmic reticulum Ca^2+^ adenosine triphosphatase 2a (SERCA2a), β1 adrenergic receptor (β_1_AR), muscarinic M2 acetylcholine receptor, mitochondrial M7, and cardiac troponin I (cTNI) in DCM patients and CVB3-infected mice [14,15,16]. However, it is to be noted that the prevalence of autoantibodies in DCM patients varies. For example, it has been reported that only 30% of patients with DCM may have anti-Myhc antibodies [17], 31% have anti-β_1_AR antibodies [18], and 20.4% have anti-cTNI antibodies [19]. These observations raise the question of whether the complete breadth of autoantibodies has been sufficiently characterized to determine their pathological significance. To this end, we used Phage ImmunoPrecipitation Sequencing (PhIP-Seq) to comprehensively analyze the autoantibody repertoire in the CVB infection model in A/J mice. We utilized a 56 amino acid (aa) long overlapping peptide library designed to redundantly cover the open reading frames of the reference mouse genome. Our analysis revealed autoantibodies to 32 novel, ubiquitously present peptides that have not been previously reported. Furthermore, we noted that the antibody reactivity patterns between CVB3 and CVB4 were similar to each other but not to influenza virus infection, highlighting the specificity of PhIP-Seq technology in discovering autoantigens.

## 2. Materials and Methods

### 2.1. Mice

Six- to eight-week-old male A/J mice and ten-week-old female BALB/c mice were purchased from the Jackson Laboratory (Bar Harbor, ME, USA) and maintained according to the institutional guidelines of the University of Nebraska-Lincoln (UNL), Lincoln, NE, USA (protocol numbers, 2097, 2107 and 2158). Approval for animal studies was granted by the Institutional Animal Care and Use Committee, UNL, and infection studies were carried out according to biosafety II guidelines. Animals were euthanized by CO_2_ asphyxiation and cervical-spinal dislocation according to the American Veterinary Medical Association guidelines.

### 2.2. Peptides and Proteins

Full-length proteins for CVB3 viral protein 1 (VP1), murine cytochrome C oxidase assembly factor 4 homolog (COA4), murine phosphoinositide-3-kinase adaptor protein 1 (PIK3AP1), protein phosphatase 1 regulatory inhibitor subunit 14C (PPP1R14C) (GenScript, Piscataway, NJ, USA), murine fibrinogen-like protein 2 (FGL2) (R&D systems, Minneapolis, MN, USA), and keyhole limpet hemocyanin (KLH) (Sigma-Aldrich, St. Louis, MO, USA) were commercially procured. Similarly, COA4 1–19 (MSTSVPQGHNWTRPVKKDD), COA4 37–57 (SHFAVQECMAQHQDWRQCQP), CVB3 VP1 39–57 (TSQVVPSDTMQTRHVKNYH), CVB3 VP1 (295-310 DYRVVNRHSATSADWQNCVW), SERCA2a 971–990 (Ac-KISLPVILMDETLKFVARNY), bovine ribonuclease (RNase) 43–56 (VNTFVHESLADVQA) (GenScript, Piscataway, NJ, USA), and Myhc-α 334–352 (DSAFDVLSFTAEEKAGVYK) (Neopeptide, Cambridge, MA, USA) were synthesized by 9-fluorenylmethyloxycarbonyl chemistry. The purity of peptides was ascertained to be more than 90% via high-performance liquid chromatography, and their identity was confirmed by mass spectroscopy. Proteins and peptides were dissolved in ultra-pure water or 1× phosphate-buffered saline (PBS) and stored in aliquots at −20 °C until further use.

### 2.3. Infection Studies

#### 2.3.1. CVB Infections

CVB3 (human isolate, Nancy strain) and CVB4-E2 were grown in Vero cells and titered as previously described [20,21]. Virus stocks were diluted in 1× PBS, and virus inocula were administered intraperitoneally (i.p) to A/J mice at doses of 10,000 TCID_50_/200 µL of CVB3 and 2000 TCID_50_/200 µL of CVB4. Similarly, the CVB3 mutant 10 (Mt10) vaccine virus grown in Vero cells was used in some experiments at a dose of TCID_50_ 0.5 × 10^6^/200 µL in A/J mice, i.p. Mice were housed in filter-top cages assembled with closed-air circulation and provided a chow diet and water; cages were changed every three days until the end of the experiment. Trans gel diet (ClearH2O, Portland, ME, USA) was provided as an alternative food and fluid source to sick mice, as needed. At termination on day 21 post-infection, animals were euthanized, and blood and tissues were harvested.

#### 2.3.2. Influenza a Infection

BALB/c mice were anesthetized with a mixture of ketamine (18.5 mg/mL) and xylazine (1.5 mg/mL) and intranasally infected with the median-lethal dose of mouse-adapted A/Puerto Rico/8/34 (NR-28652) Influenza A virus. Body weights were recorded daily, and animals that reached 75% of their initial weights were euthanized; sera were collected on day 0 and day 21 at termination and stored at −20 °C.

### 2.4. Immunization Procedures

A/J mice were subcutaneously immunized on days 0 and 7 in the inguinal and sternum regions, with Myhc-α 334–352 or SERCA2a 971–990 emulsified in complete Freund’s adjuvant (CFA) containing *Mycobacterium tuberculosis* H37RA extract (5 mg/mL; Difco Laboratories, Detroit, MI, USA). Pertussis toxin was administered on days 0 and 2 (100 ng/mouse, i.p) (List Biological Laboratories, Campbell, CA, USA). Sera were collected on day 0 and day 21 post-immunization at termination.

### 2.5. Phage ImmunoPrecipitation Sequencing (PhIP-Seq)

#### 2.5.1. VirScan, Human, and Mouse Peptide Library Design and Cloning

The design, construction, and use of the VirScan and human peptidome libraries have been described elsewhere [22,23]. The design and cloning of the mouse peptide library followed previously published PhIP-Seq protocols [24]. After downloading the mouse proteome (UP000000589) from the UniProt database (accessed on 1 January 2018), mouse proteins KV3A6_MOUSE and KV3A7_MOUSE were excluded because of degeneracy issues. The proteome of 49,734 unique protein sequences was then compressed into a de Bruijn graph using 20-mer peptides, and 56-mer tiles were sampled from the graph to generate the peptide library comprising 419,915 peptides. All C-terminal tiles were manually added to the pool. The peptide tiles were reverse-translated into their corresponding DNA sequences and recoded by introducing silent mutations to remove any instances of EcoRI or HindIII restriction sites, which were utilized for cloning. PCR primer binding sequences AGGAATTCCGCTGCGT and ATGGTCACAGCTGTGC were added to the 5′ end and the 3′ end, respectively, bringing the total oligonucleotide length to 200 bases. These encoding oligonucleotides were synthesized (Twist Bioscience, South San Francisco, CA, USA) and cloned into the mid-copy T7-FNS2 phage display vector as previously described [25] and packaged using the T7 Select Packaging Kit (EMD Millipore Sigma, Burlington, MA, USA). Library quality assessment was performed as previously described. All design operations were implemented in the pepsyn Python package [24].

#### 2.5.2. Mouse Serum Antibody Profiling

The standard protein A/G PhIP-Seq protocol, which was employed here, has been described in detail [24]. Briefly, 20 µL of serum diluted 1:100 in 1× PBS was used as input for each immunocapture (immunoprecipitation, IP) reaction. The mouse peptide library was mixed with diluted serum at 10^5^-fold library coverage. The mixture was allowed to rotate overnight at 4 °C, followed by a 4 h IP with protein A and protein G coated magnetic beads (Dyna beads, Invitrogen, Waltham, MA, USA). A first PCR was performed with primers that flank the displayed peptide inserts. A second PCR added adapters and indexes for Illumina sequencing. Fastq files were aligned to obtain read count values for each peptide in the library, followed by detection of antibody reactivity by comparison with a set of mock IPs (no antibody input) run on the same plate. For a peptide to be considered reactive, the sequencing read counts needed to exceed 15, the fold-change compared to the mock IPs needed to be at least 5, and the *p*-value of differential abundance needed to be below 0.001 (fold-changes and *p*-values were calculated using the EdgeR software).

### 2.6. Indirect Enzyme-Linked Immunosorbent Assay (ELISA)

Proteins and peptides were diluted in 1× coating buffer (eBioscience, San Diego, CA, USA) (5 ug/mL) and coated onto 96-well polystyrene high-binding microtiter plates. After incubation at 4 °C overnight, plates were washed with 1× PBS/0.05% Tween-20 and blocked with 1× PBS/2% BSA/5% normal goat serum for 1.5 h at RT. Diluted serum samples were then added in duplicates. The plates were incubated at 37 °C for 1 h and washed. Horseradish peroxidase (HRP)-labeled goat anti-mouse total immunoglobulin (Ig), IgM, IgG1, IgG2a, IgG2b, IgG3, and IgA were added as secondary antibodies (Southern Biotech, Birmingham, AL, USA). After incubation at 37 °C for 1 h, the plates were washed and 1× tetramethylbenzidine solution was added as a substrate (Rockland Immunochemicals, Pottstown, PA, USA). The reactions were then stopped using 1M phosphoric acid, and plates were read at 450 nm using an automated ELISA reader (BioTek Instruments, Winooski, VT, USA) to measure the optical density (OD) values.

### 2.7. Statistics

The data obtained from PhIP-Seq were subjected to Barnard’s exact test to determine significant differences between infected and naïve groups. To analyze total Ig and antibody isotypes, one-way ANOVA with Tukey’s test or two-way ANOVA with Tukey’s test/Sidak’s post-test were used. Statistical analyses were performed using GraphPad Prism software v8.0 (GraphPad Software, Inc. La Jolla, CA, USA).

## 3. Results

### 3.1. VirScan Screening of Sera from CVB3-Infected Mice Revealed Antibody Reactivity to CVB3 with a High Degree of Specificity and Sensitivity

**Figure 1:**
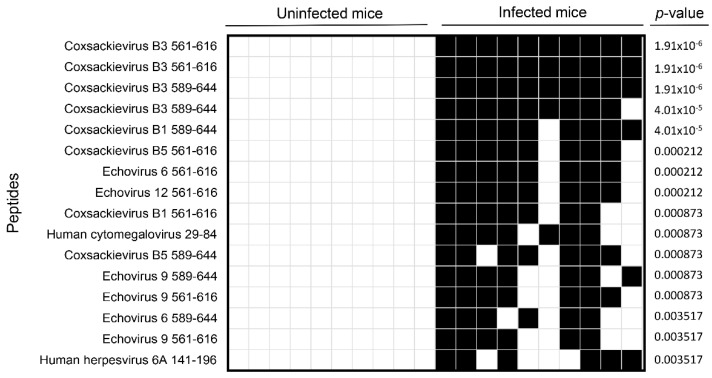
Viral peptides identified by VirScan. Sera collected from CVB3-infected or control animals were subjected to PhIP-Seq as described in the methods section. Differential abundance of antibody reactivities to viral peptides were determined in the corresponding groups using the EdgeR software, and the data were compared between groups using Barnard’s exact test. Each column represents one animal. Blank and filled squares represent absence and presence of antibody reactivities, respectively (*n* = 10 animals per group).

Although our primary goal was to investigate the autoantibody repertoire in CVB3 myocarditis, we first used the human virome peptide library, VirScan, expecting CVB3-infected animals to show reactivity specifically to CVB3 viral proteins. Essentially, the VirScan library consists of T7 bacteriophage expressing 56 aa peptide tiles with a 28 aa overlap that together span the coding sequence of all human viruses. Upon mixing this bacteriophage display library with sera, the antibody-bound sequences are separated by immunocapture and sequenced to determine the identities of reactive viral peptides [22,24,26]. Our analysis revealed antibody reactivities mainly to CVB3 peptides in all infected animals with a high degree of specificity and sensitivity (Figure 1). While similar reactivity was noted for CVB1 (70–90%) and CVB5 (70–80%) peptides, lower reactivities were observed for peptides from echoviruses (60–80%) (Figure 1). Further, comparison of CVB3 with the PhIP-Seq reactive peptides of CVB1 and CVB5 revealed 70 to 91% sequence similarity, suggesting that antibody cross-reactivity among closely related serotypes is detectable using VirScan. Similar sequence comparisons between CVB3 and reactive peptides of echoviruses revealed varying levels of similarity (Echovirus 6, 63–80%; Echovirus 9, 68–91%; and Echovirus 12, 71%)**.** Such data were not noted in naïve mice, suggesting the presence of additional cross-reactive antibodies, likely directed against unrelated environmental antigens. Taken together, the data indicate that the PhIP-Seq methodology yields reliable information to identify antibodies with a high degree of specificity and sensitivity.

### 3.2. Analysis of the Mouse Peptide Library Revealed Antibodies to Novel Proteins That Were Previously Unknown in CVB3 Myocarditis

We utilized PhIP-Seq to screen sera harvested from animals infected with CVB3 or control groups, using the mouse peptide library containing 419,959 peptides with a length of 56 aa each. Of note, acute myocarditis induced by CVB3 is associated with detectable viruses that may last for 14 to 18 days, beyond which infectious virions become undetectable, but inflammation persists, and the affected animals develop DCM features over a period of 30 to 90 days post-infection [13,27]. We chose day 21 to screen our serum samples using PhIP-Seq because reports indicate that autoantibodies to some of the known antigens, such as Myhc-α, ANT, and BCKDk, were detected in sera from CVB3-infected animals during the 15- to 28-day post-infection period [10].

**Figure 2:**
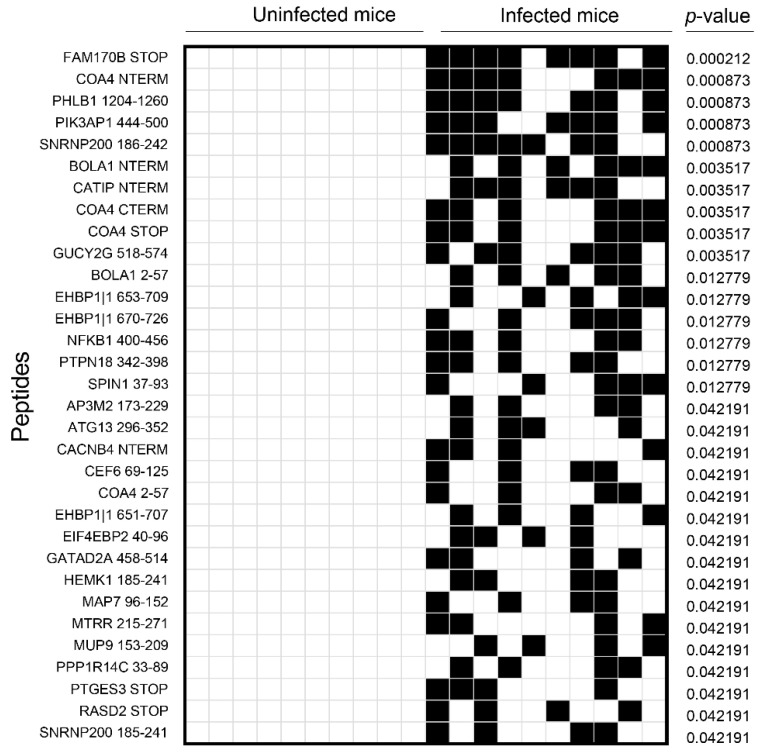
Mouse proteins identified by PhIP-Seq. Sera collected from CVB3-infected or control animals were subjected to PhIP-Seq using the mouse peptide library as described in the methods section. Differential abundance of antibody reactivities to mouse peptides was determined in the corresponding groups using the EdgeR software, and the data were compared between groups using Barnard’s exact test. Each column represents one animal. Blank and filled squares represent absence and presence of antibody reactivities, respectively (*n* = 10 animals per group).

PhIP-Seq analysis revealed significant antibody reactivity to 32 previously unreported peptides with varied prevalence (40 to 80%) (Figure 2). Notably, such reactivity was lacking in naïve mice, implying that CVB3 infection is required for producing these autoantibodies. Furthermore, while antibody reactivities for most proteins occurred for a single peptide sequence of 56 residues, reactivity to COA4 was noted for four overlapping peptides covering the entire length of the protein (Figure S1), indicating that COA4 antibodies produced in CVB3 infection may have multiple epitopes that reflect a polyclonal, antigen-driven immune response (Figure 2). Surprisingly, however, PhIP-Seq antibody reactivities for known antigens were low, but when detected, they were not significantly different between groups. For example, only 10% of animals in both infected and naive groups reacted to Myhc-α/Myhc-6, whereas 40% of CVB3-infected animals showed reactivity to Myhc-β/Myhc-7 as opposed to 10% of naïve mice, but the two groups showed no difference in reactivity to cTNI (50% each) (Table S1). However, since PhIP-Seq led to the detection of autoantibodies to several ubiquitously expressed antigens, we sought to determine whether serum from CVB3-infected animals could react to human proteins, since mice and humans have ~85% similarities in their protein-coding sequences [28,29]. To that end, we analyzed the same samples using PhIP-Seq with the human peptide library containing 274,206 sequences (Figure S2). The analysis revealed antibody reactivity to peptides from 10 proteins in the infected group (ranging from 40–80%), which significantly differed from reactivity in naïve mice. While these data informed us of the potential significance of novel antigens in CVB3 myocarditis, it was important to confirm such candidates by secondary assays.

### 3.3. ELISA Analysis of Serum Samples from CVB3-Infected Animals Validated the Results Obtained with PhIP-Seq

To confirm the data obtained from PhIP-Seq, we utilized ELISA to detect the presence of autoantibodies to four candidate antigens. We selected COA4 and PPP1R14C because their antibodies were detected in the infected group with overlapping reactivity in both mouse and human peptide libraries, although only 4 out of 10 animals had PPP1R14C-reactive antibodies (Figure 2 and Figure S2). On the other hand, PIK3AP1 was selected because 70% of infected animals had antibodies to PIK3AP1 peptides, and its relevance has been noted in autoimmunity [30] (Figure 2). We selected FGL2 although differences between infected and naïve groups were only marginally significant (data not shown), expecting the ELISA results to correlate with data from PhIP-Seq. In this direction, we segregated the serum samples subjected to PhIP-Seq analysis into infected/PhIP-Seq^+^ and infected/PhIP-Seq^−^ groups and compared their profiles with those of naïve mice. While samples from infected animals categorized as PhIP-Seq^+^ were positive for COA4 or PIK3AP1, the PhIP-Seq^−^ samples were negative for antibodies to either of the two proteins. In all these analyses, we used CVB3 VP1 and KLH as positive and negative controls, respectively, and performed indirect ELISAs using the proteins expressed in *E. coli* and measured antibody reactivities based on OD values.

**Figure 3:**
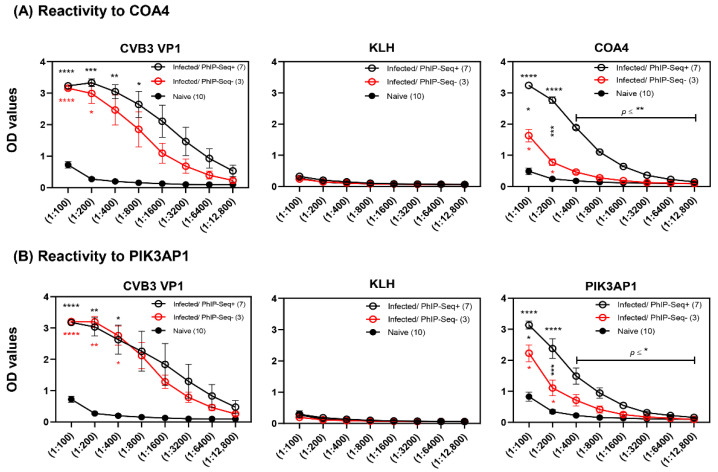
Sera from CVB3-infected mice reveals antibodies to COA4 and PIK3AP1. Sera were collected from A/J mice that were infected with CVB3 (*n* = 10) or control groups (*n* = 10) on day 21 and evaluated by ELISA for the presence of COA4- and PIK3AP1-reactive antibodies. (**A**) Reactivity to COA4. Sera from infected animals positive (infected/PhIP-Seq^+^) or negative (infected/PhIP-Seq^−^) for COA4, along with sera from naïve mice, were serially diluted and added in duplicates to plates coated with COA4, CVB3 VP1 (positive control), and KLH (negative control). (**B**) Reactivity to PIK3AP1. Sera from infected animals positive (infected/PhIP-Seq^+^) or negative (infected/PhIP-Seq^−^) for PIK3AP1, along with sera from naïve mice, were serially diluted and added in duplicates to plates coated with PIK3AP1, CVB3 VP1 (positive control), and KLH (negative control). Sequentially, anti-mouse HRP-conjugated total Ig was added as detection antibody followed by substrate. After stopping the reactions, plates were read at 450 nm to obtain OD values. Mean ± SEM values are shown. Two-way ANOVA with Tukey’s test was used to determine significance between groups. * *p* ≤ 0.05, ** *p* ≤ 0.01, *** *p* ≤ 0.001, and **** *p* ≤ 0.0001.

First, we noted that sera from infected but not control animals reacted to CVB3 VP1 as demonstrated by detection of total Ig (Figure 3A,B, left panels). Such reactivity was lacking in naïve animals, as expected. Likewise, none of the samples reacted to KLH, an irrelevant antigen, indicating the antigen specificity of CVB3 VP1-reactive antibodies (Figure 3A,B, middle panels). Second, total Ig for COA4 or PIK3AP1 were detected in both infected/PhIP-Seq^+^ or infected/PhIP-Seq^−^ groups, but their levels tended to be higher in the former than the latter (Figure 3A,B, right panels). Expectedly, sera from naïve mice did not react to either protein. While these observations indicated that the data obtained from PhIP-Seq and ELISA analyses complemented each other, the finding that higher reactivity in infected/PhIP-seq^+^ groups relative to infected/PhIP-seq^−^ groups suggested that PhIP-Seq may have missed conformational epitopes. Third, similar comparisons for PPP1R14C revealed reactivity, but differences between CVB3-infected and naïve animals were not significant (Figure S3, left panel). This could be because differences between groups by PhIP-seq were only marginally significant in the mouse peptide library (*p* = 0.042) (Figure 2). Similarly, lack of reactivity to FGL2 in ELISA was expected (Figure S3, right panel), which agreed with PhIP-seq and revealed no significant differences (data not shown). Overall, the antibody reactivity patterns as analyzed by ELISA agreed with that from PhIP-Seq for COA4 and PIK3AP1. Nonetheless, since strong antibody reactivities were noted for these proteins, we sought to investigate their isotypes.

### 3.4. Antibodies to COA4 and PIK3AP1 Detected in CVB3-Infected Animals Were Predominantly of the IgG2a Isotype

**Figure 4:**
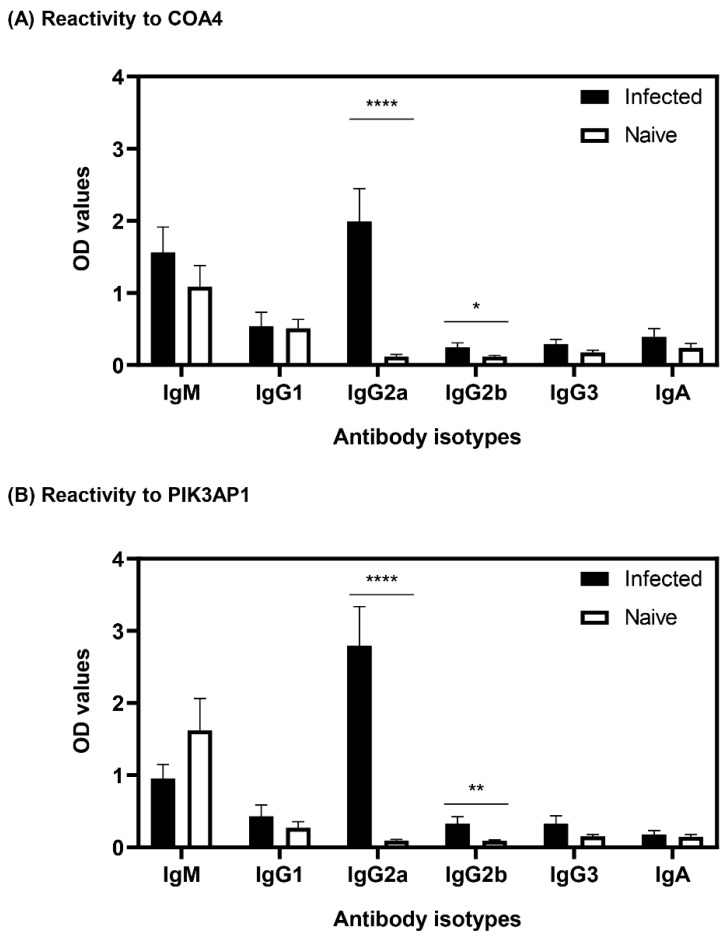
Antibody reactivities to COA4 and PIK3AP1 in CVB3-infected animals were predominantly of IgG2a. (**A**,**B**) Reactivity to COA4 and PIK3AP1. Sera harvested from A/J mice infected with or without CVB3 were diluted (1:100) and added in duplicates to plates coated with COA4 or PIK3AP1. After adding HRP-conjugated anti-mouse IgM, IgG1, IgG2a, IgG2b, IgG3, and IgA as detection antibodies and substrate, reactions developed after the addition of substrate were stopped, and the plates were read at 450 nm to obtain OD values. Mean ± SEM values representing three samples per group with each sample representing a pool of sera from 3–4 mice. Two-way ANOVA with Sidak’s test was used to determine significance between groups. * *p* ≤ 0.05, ** *p* ≤ 0.01, **** *p* ≤ 0.0001.

We determined the antibody isotypes for COA4 and PIK3AP1 by using sera harvested from CVB3-infected and naïve mice independent of the samples used for PhIP-Seq. The ELISA data revealed that sera from the CVB3-infected group contained IgG2a antibodies for both COA4 and PIK3AP1, but controls did not (Figure 4A,B). Although similar trends were seen for IgG2b, the levels were low, and other isotypes (IgM, IgG1, IgG3, and IgA) were not significantly different. Additionally, by using CVB3 VP1 as a positive control, we noted that the sera from CVB3-infected but not naïve animals had multiple IgG isotypes (IgG1, IgG2a, IgG2b, and IgG3) and a relatively low amount of IgA but not IgM (Figure S4). Previous studies have shown that interferon (IFN)-γ mediates isotype switching to IgG2a [31,32], and in the past, we have demonstrated that the serum from CVB3-infected animals revealed the presence of IFN-γ, in addition to relatively low levels of interleukin (IL)-2, IL-10, IL-6, IL-17A, and tumor necrosis factor (TNF)-α [33,34]. The selective induction of IgG2a for self-antigens (COA4 and PIK3AP1) as opposed to broad IgG responses to foreign (virus) antigens may reflect the nature of antigens and their ability to induce T cell responses. Together, our data suggested that CVB3 infection leads to the production of autoantibodies to COA4 and PIK3AP1 but raised a question whether such reactivity could be seen in infections caused by other CVB serotypes. 

### 3.5. Sera from CVB4-Infected Animals Showed Antibodies to COA4 and PIK3AP1 with Profiles Similar to Those of CVB3 Infection

**Figure 5:**
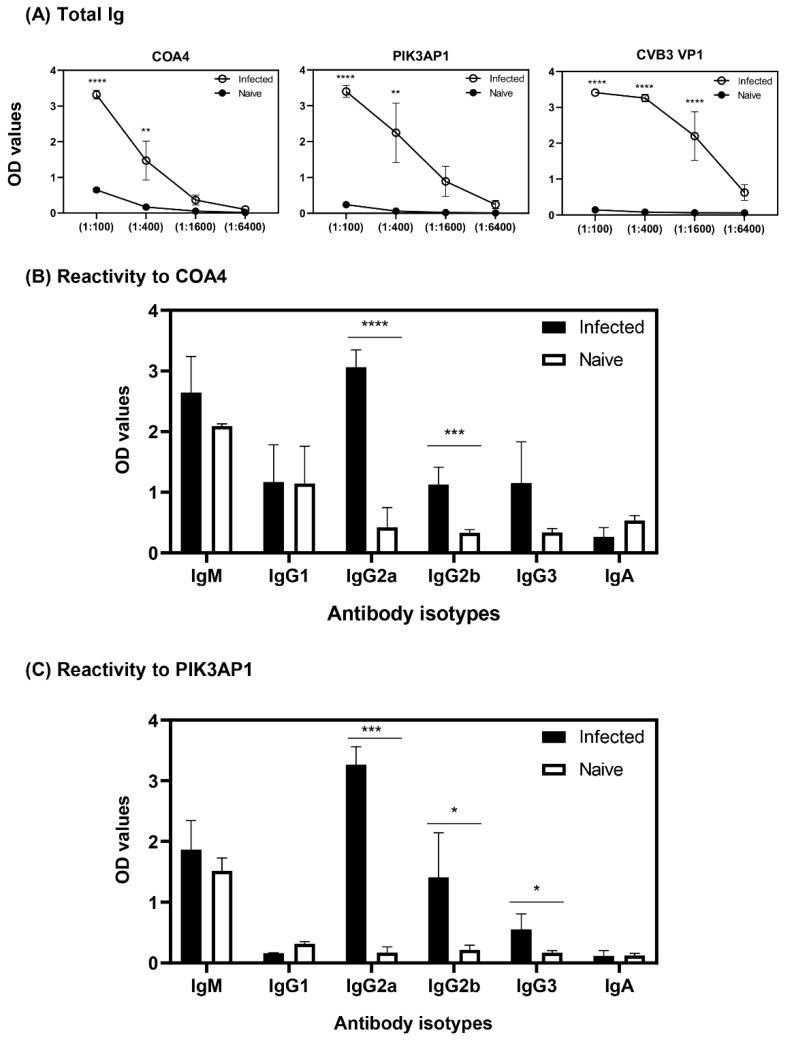
Sera from CVB4-infected animals revealed autoantibodies to COA4 and PIK3AP1 similar to CVB3 infection. (**A**) Total Ig. A/J mice were infected with or without CVB4, and sera collected at termination on day 21 post-infection were serially diluted and added in duplicates to plates coated with COA4, PIK3AP1, or CVB3 VP1. After washing and addition of HRP-conjugated anti-mouse total immunoglobulin and substrate, reactions were stopped, and the OD values were measured at 450 nm. (**B**,**C**) Reactivity to COA4 and PIK3AP1. The serum samples described above were evaluated for their reactivity to the indicated proteins. Diluted serum samples (1:100) were first added, followed by HRP-conjugated anti-mouse IgM, IgG1, IgG2a, IgG2b, IgG3, and IgA as detection antibodies. After adding substrate, reactions were stopped to measure the OD values. Mean ± SEM values for infected (*n* = 4) and naïve groups (*n* = 3), each representing the pool of sera from 3 to 4 mice, are shown. Two-way ANOVA with Sidak’s test was used to determine significance between groups. * *p* ≤ 0.05, ** *p* ≤ 0.01, *** *p* ≤ 0.001, and **** *p* ≤ 0.0001.

Six CVB serotypes (1 to 6) exist, and multiple serotypes could induce similar diseases, as noted with CVB3 and CVB4. While both serotypes can cause myocarditis, the disease severity is relatively higher with CVB3 than CVB4, but both induce pancreatitis [35,36]. To determine whether COA4- and PIK3AP1-reactive antibodies are specific to CVB3 or whether their production can be expected in other serotype infections, we used sera from CVB4-infected mice for ELISA. As shown in Figure 5A, the total Ig levels reacting to COA4, PIK3AP1, and CVB3 VP1 were evident only in the CVB4-infected animals but not in controls. Further evaluation revealed mainly IgG2a, and to a lesser extent, IgG2b, for both COA4 and PIK3AP1 in the CVB4-infected group (Figure 5B,C), and this pattern is identical to that found in CVB3-infected mice (Figure 4). Given these similarities, we believe that the pathogenetic pathways for the induction of IgG2a antibodies for COA4 and PIK3AP1 may be similar in CVB3 and CVB4 infections. While such a proposition could be true because of the high degree of similarity between CVB3 and CVB4 (92%) sequences and because both serotypes produce similar diseases, we tested an unrelated virus to ascertain virus specificity for the induction of COA4 or PIK3AP1 antibodies.

### 3.6. Mice Infected with the Influenza Virus Did Not Reveal Detection of COA4 or PIK3AP1 Antibodies

**Figure 6:**
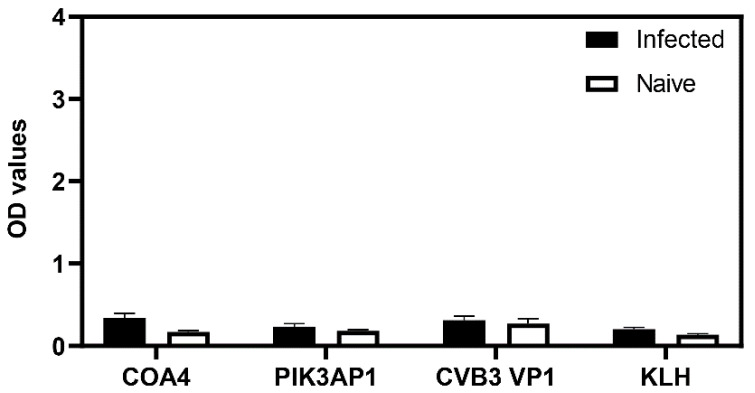
Infection with influenza virus does not lead to the production of antibodies to COA4 or PIK3AP1. BALB/c mice were infected with or without mouse-adapted influenza A virus (A/Puerto Rico/8/34 (NR-28652)). Sera collected at termination on day 21 post-infection were diluted (1:100) and tested for their reactivity to COA4, PIK3AP1, CVB3 VP1, or KLH. After adding HRP-conjugated anti-mouse total Ig and substrate, reactions were stopped to measure the OD values at 450 nm. Mean ± SEM values represent three samples in each group. Two-way ANOVA with Sidak’s test was used to determine significance between groups.

To establish a virus control system for CVB infections, we performed an experiment to infect groups of BALB/c mice that are susceptible to influenza infection [37,38]. Sera collected from infected or naïve animals on day 21 post-infection were analyzed for their reactivity to COA4 and PIK3AP1, with CVB3 VP1 and KLH as controls. The data indicated no reactivities to any proteins tested in the infected or control groups (Figure 6). Lack of antibody reactivity to COA4 and PIK3AP1 in influenza virus infection, but the presence of COA4 and PIK3AP1 antibodies in CVB3 and CVB4 infections, may imply that they may be released from damaged heart or pancreatic tissues, triggering an autoimmune response.

### 3.7. Virulent CVB3 Is Necessary to Induce COA4- or PIK3AP1-Reactive Antibodies

**Figure 7:**
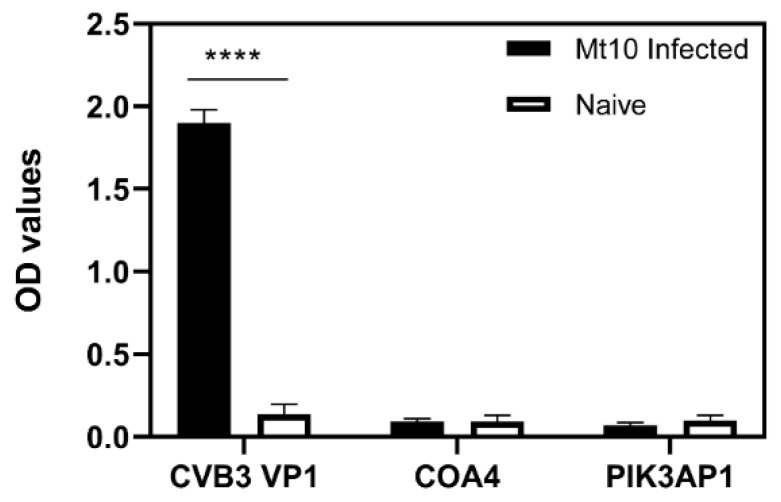
Appearance of autoantibodies to COA4 and PIK3AP1 requires infection with a virulent virus. A/J mice were infected with a modified live attenuated Mt10 CVB3 vaccine virus or saline, and after 21 days, serum samples were collected. Diluted serum samples (1:100) were added to plates coated with CVB3 VP1, COA4, or PIK3AP1. After incubation and addition of HRP-conjugated anti-mouse total Ig and substrate, reactions were stopped to measure the OD values at 450 nm. Mean ± SEM values in each group (*n* = 3) are shown, with each sample representing the pool of sera from 3 to 4 mice. One-way ANOVA with Tukey’s test was used to determine significance between groups. **** *p* ≤ 0.0001.

To test the hypothesis that the tissue destruction resulting from CVB infection is essential for inducing autoantibodies to COA4 or PIK3AP1, we used the modified live attenuated strain of CVB3, designated Mt10, which we had recently identified as a vaccine candidate. The Mt10 virus infects the heart and pancreas but does not cause myocarditis or pancreatitis [21]. Sera collected from mice infected with the Mt10 virus were analyzed by ELISA for antibodies to CVB3 VP1 (positive control), COA4, and PIK3AP1 (test antigens). Expectedly, CVB3 VP1-reactive antibodies were present in Mt10-infected mice (Figure 7). In contrast, antibody reactivity was absent for both COA4 and PIK3AP1, indicating that tissue destruction is necessary for the production of either autoantibody but raising a question as to the sources for the release of the above antigens.

### 3.8. The Presence of COA4-Reactive Antibodies Was Revealed in Animals Immunized with Myhc-α 334-352

**Figure 8:**
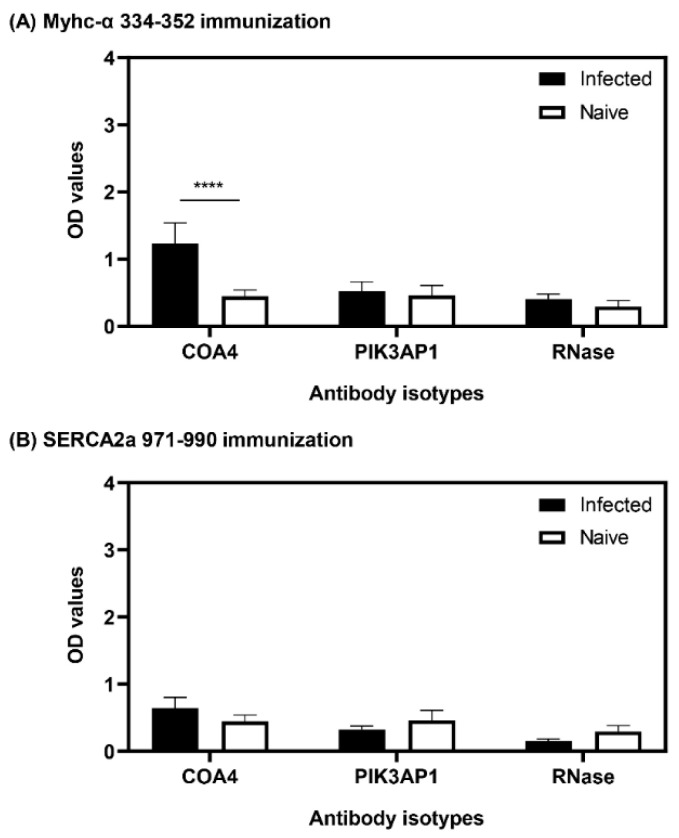
Animals immunized with Myhc-α 334–352 reveal the presence of autoantibodies to COA4. (**A**,**B**) Myhc-α 334–352 and SERCA2a 971–990 immunization. A/J mice were immunized with Myhc-α 334–352 or SERCA2a 971–990 in CFA emulsions or saline, and sera were collected 21 days later at termination. Diluted serum *samples* (1:100) were added to plates in duplicates coated with COA4, PIK3AP1, or RNase 43–56 (control). After addition of HRP-conjugated anti-mouse total Ig as the detection antibody, reactions were stopped, and OD values were then measured at 450 nm. Mean ± SEM values representing six samples per group are shown. Two-way ANOVA with Sidak’s test was used to determine significance between groups. **** *p* ≤ 0.001.

To further investigate whether tissue damage is critical for producing COA4 and PIK3AP1 antibodies, we used the autoimmune myocarditis models induced with Myhc-α 334–352 [39] or SERCA2a 971–990 [40]. Essentially, both antigens induce myocarditis, with severity being relatively more widespread with Myhc-α 334–352 than SERCA2a 971–990, but the latter induces mainly atrial myocarditis [40,41]. We collected sera on day 21 post-immunization for ELISAs. We noted a marginal increase in antibodies to COA4 but not to PIK3AP1, which occurred only in the Myhc-α 334–352-immunized group (Figure 8). The reason for the differences noted between the two immunization models is unknown. However, given the low antibody reactivity to COA4 occurring only at 1:100 dilutions in the Myhc-α 334–352-immunization model, it is difficult to interpret whether the heart is, in fact, the source for COA4 release that enables induction of autoantibodies. However, other potential autoimmune mechanisms cannot be discounted. 

### 3.9. The Generation of COA4-Reactive Antibodies Did Not Involve Cross-Reactivity to CVB Proteins

We investigated whether mimicry epitopes, if any, in the CVB3 viral proteins could contribute to the induction of autoantibodies to COA4 or PIK3AP1 by cross-reactivity. To address this question, we analyzed the sequences for COA4 and PIK3AP1 used in PhIP-Seq, noting 30–40% similarity in two stretches of aa between CVB3 VP1 and COA4. Such mimicry sequences were not found for PIK3AP1. We then synthesized two pairs of mimicry epitopes (20-mers), CVB3 VP1 39–57/COA4 1–19 and CVB3 VP1 295–310/COA4 37–57, and subjected them to ELISA, along with the full-length CVB3 VP1/COA4 (positive control) and KLH/RNase (negative control), using sera from naïve or CVB3-infected mice (Figure S5). Expectedly, sera from naïve mice did not react with any of the test antigens indicated above (Figure S5, top panel). In contrast, sera from CVB3-infected mice responded to the full-length proteins of CVB3 VP1 or COA4 but not KLH or RNase 43–56, which was also expected. However, the reactivity for mimicry epitopes was completely lacking in the CVB3-infected group, reducing the likelihood that molecular mimicry underlies the appearance of autoantibodies to COA4.

## 4. Discussion

Here, we report the identification of autoantibodies to at least 32 ubiquitously expressed peptides from 25 proteins that were not previously reported in viral myocarditis. In this effort, we adopted the PhIP-Seq platform primarily involving a mouse peptide library, which permitted us to comprehensively analyze autoantibody repertoires in the mouse model of CVB3 myocarditis. Various methods, such as peptide and cDNA arrays [42,43], phage display [44], fluid-phase immunoassays [43], and antigen microarrays [45], have been used in discovering autoantigens. Among these, the phage display involving the T7 bacteriophage has proven to be a fast and high-throughput proteomic screening platform [24,46,47]. This approach has been widely used to screen antibodies for reactivity to viruses, autoantigens (including the neoantigens in tumor settings), microbial toxins and virulence factors [48], and allergens in basic and clinical research [23,49,50,51]. One significant advantage of using the T7 bacteriophage display is its versatility, which facilitates the detection of antibodies to a wide range of antigens while screening large libraries that contain peptides covering all open reading frames [25]. In our studies, we used VirScan, which includes peptide sequences for all known viruses, and the mouse peptide library, which consists of 419,959 peptides representing all mouse proteins. Furthermore, applying PhIP-Seq in autoimmune research has led to the identification of several novel autoantigens, as demonstrated in rheumatoid arthritis, type 1 diabetes, and inclusion body myositis among others, as well as the detection of known antigens [52,53,54,55]. Generally, pathogens are implicated as the triggers of autoimmune responses to self-antigens that can potentially be released secondary to primary insults. Consistent with this theme, we have demonstrated that viral myocarditis induced with CVB3 leads to the generation of autoreactive T cells with multiple antigen specificities as a secondary event [56,57], indicating that antibodies corresponding to such autoantigens could be produced with T cell help through cytokines. 

Antibodies to various cardiac antigens indicated above have been reported in CVB3 myocarditis [15]. The determination of antibodies for these select antigens was prompted by human studies with a potential caveat that the entire breadth of antibodies produced in response to CVB3 infection might not have been captured. Arguably, such studies in suitable animal models—in our case, CVB3 myocarditis in A/J mice—with relevance to viral cardiomyopathy may identify novel antigens of disease importance. To this end, we used PhIP-Seq on sera from CVB3-infected mice and compared their profiles with those of healthy mice, leading us to make several observations.

First, using VirScan, we tested the hypothesis that virus-reactive antibodies produced by CVB3-infected animals should be detected virus-specifically by PhIP-Seq, and our data support this proposition (Figure 1). Such an analysis may also lead to the detection of cross-reactive antibodies for closely related serotypes, as we have noted with CVB1 and CVB5. However, a low degree of cross-reactivity seen for other viruses may be regarded as a cross-reactivity of antibodies directed against other environmental antigens.

Second, we applied PhIP-Seq to interrogate the mouse peptide library containing more than 400,000 peptides for various proteins. While this analysis led us to note antibodies for 32 peptides that are ubiquitously expressed (Figure 2), antibodies for known antigens were low or absent except for (Myhc-7/Myhc-β) (Table S1). Such discrepancies are not surprising because the detection of antibodies to autoantigens such as Myhc-α, SERCA2a, β_1_AR, ANT, and cTNI were previously made based on traditional assays, such as ELISA, using whole proteins [10,58], immunofluorescence [40,59] or immunohistochemistry [60,61], as opposed to the shorter peptide sequences in PhIP-Seq. Although it is generally thought that PhIP-Seq does not detect antibodies to post-translationally modified proteins or conformational epitopes [24,62], small peptide fragments can also bear epitopes [63,64]. We next asked whether sera from CVB3-infected animals could yield antibody-reactivity patterns similar to those in the mouse peptide library by screening the human peptide library. The data revealed significant reactivity to 10 proteins, with COA4 being the strongest candidate (Figure S2). By comparing sequence similarities between human COA4 and murine COA4, we noted the identities of proteins between the two to be more than 90% identical, including a stretch of 23 aa (COA4 33–56) to be 100% identical, a potential reason for the cross-recognition described above (Figure S1).

Third, we sought to confirm the data obtained from PhIP-Seq by ELISA as a secondary assay, expecting their evaluations to agree with each other, and for this, we selected COA4 and PIK3AP1. We found that the sera from CVB3-infected mice consistently reacted to COA4 (Figure 3), which is a mitochondrial protein, in both mouse and human peptide libraries with high specificity and sensitivity, indicating that COA4-reactive antibodies may be relevant to CVB3 pathogenesis. In support of this proposition, antibodies for other mitochondrial proteins, such as ANT [65], BCKDk [10,66], pyruvate dehydrogenase [67], and mitochondrial M7 [68], have been reported in DCM patients and/or CVB3 myocarditis. Additionally, we previously demonstrated that CVB3 infection can lead to the induction of ANT-reactive T cells localized in the liver with a potential for recirculation back to the heart under inflammatory conditions [56]. Likewise, anti-mitochondrial antibodies have been reported in systemic lupus erythematosus (SLE) [69], primary biliary cholangitis [70], rheumatoid arthritis [71], and other diseases. Since CVB3 is a lytic virus, necrotic events resulting from CVB3-induced damage may facilitate the release of intracellular antigens, including mitochondrial proteins, leading to the generation of their autoantibodies [56]. Likewise, in combination with autophagy, the resident antigen-presenting cells may take up dead or dying cells and present their antigens to lymphocytes in the draining lymph nodes [72,73]. Similarly, anti-PIK3AP1 antibodies may also have a role in the development of myocarditis, since PIK3AP1 functions as an adaptor protein involving the PI3K signaling pathway [74,75], and PIK3AP1 has been reported to have a role in SLE [30]. Overall, because the antibody profiles obtained from both PhIP-Seq and ELISA analyses complemented each other for COA4 and PIK3AP1, we decided to further characterize their antibodies.

In that direction, we noted that the antibody isotypes for both COA4 and PIK3AP1 mainly represented the IgG2a isotype (Figure 4), indicating that the T cells specific to these antigens might have been generated in the CVB3-infected animals. However, we have not investigated this possibility. Further evaluation of antibodies for COA4 and PIK3AP1 in CVB4 infection led us to note similar profiles, but not in influenza virus infection, indicating the existence of common pathogenetic pathways in both CVB3 and CVB4 infections. We propose that virus-induced tissue damage is critical for generating autoantibodies to COA4 and PIK3AP1, as animals infected with an avirulent virus did not reveal these antibodies (Figure 6). It is possible that the potential source for COA4 could be the heart, since autoimmune myocarditis induced with Myhc-α led to the detection of COA4, albeit at a low level. Nonetheless, we ruled out molecular mimicry hypothesis as a possible mechanism for detecting COA4-reactive antibodies through cross-reactivity. Taken together, our data suggest that the generation of autoantibodies for COA4 and PIK3AP1 requires active CVB infections but raise questions as to their significance.

Autoantibodies can be generated in response to the release of subcellular antigens due to infections [76]. While autoantibodies could play a pathogenic role in exacerbating the disease, they can also be used as biomarkers. Pathogenically, autoantibodies can participate in disease pathogenesis through antibody/complement-mediated inflammation [77] or macrophage activation [78] or be independent of inflammatory pathways [79]. Specifically, autoantibodies detected in DCM/inflammatory cardiomyopathy patients could be broadly classified into those reacting to membrane receptors, cellular antigens, and mitochondrial antigens [15]. Autoantibodies may promote apoptotic pathways leading to cardiac remodeling [79] or may alter the enzymatic/signaling pathways as demonstrated with β_1_AR-reactive antibodies that modulate the functions of mitochondrial proteins, as shown with ANT [79]. Currently, we do not know the significance of the autoantibodies detected in viral myocarditis, but their importance as biomarkers cannot be ruled out. This notion is supported by the fact that the detection of autoantibodies does not necessarily mean that autoimmune disease ensues [80,81,82]. In fact, a theme is developing that the determination of autoantibodies in combination with other multi-analyte ‘omic’ profiles may be helpful for predicting the diseases as well as disease intervention or prevention strategies [47,53,83].

In summary, we used the PhIP-seq platform to investigate the production of autoantibodies in CVB3 myocarditis as a disease model for viral cardiomyopathy. This analysis led us to note autoantibodies for several unknown antigens that are ubiquitous in nature. While our data support the notion that virus-induced damage is critical in inducing autoantibodies, the source for their release could be the heart or pancreas, as both organs are affected in CVB infections. Further, since COA4- and PIK3AP1-reactive antibodies were mainly of the IgG2a isotope, it is possible that CVB infections may also lead to the induction of antigen-specific CD4 T cells, whose help is critical in producing antibodies to protein antigens. The significance of COA4- or PIK3AP1-reactive antibodies is currently unknown, but their role can be investigated in immunization settings in the future. Finally, in light of the finding that autoantibody reactivity to COA4 was consistent in both mouse and human libraries, COA4-reactive antibodies may be relevant to viral cardiomyopathy patients because they could be used as biomarkers.

## 5. Conclusions

Using PhIP-Seq technology, we identified autoantibodies for various self-antigens that were not previously reported in viral myocarditis. Although their significance is currently unknown, infection with CVB3 was essential for the formation of autoantibodies, suggesting a possibility that they could be used as biomarkers of myocarditis with viral origins.

## Data Availability

Not applicable.

## Supplementary Materials

The following supporting information can be downloaded at: https://www.mdpi.com/article/10.3390/biology11071055/s1, Figure S1: Comparison of COA4 peptide sequences between mice and humans, and their antibody reactivities in controls and animals infected with CVB3. COA4 sequences present in mice and human peptide libraries (left panel) were compared. Antibody reactivities to each of the above are shown in the heat map corresponding to controls and CVB3-infected group (right panel). Each column represents one animal. Blank and filled squares represent absence and presence of reactivities, respectively (*n* = 10 animals per group). Box indicates similarities across all sequences. Figure S2: Human proteins identified by PhIP-Seq. Sera collected from CVB3-infected or control animals were subjected to PhIP-Seq using the human peptide library as described in the methods section. Differential abundance of antibody reactivities to human peptides was determined in the corresponding groups using the EdgeR software, and the data were compared between groups using Barnard’s exact test. Each column represents one animal. Blank and filled squares represent absence and presence of reactivities, respectively (*n* = 10 animals per group). Figure S3: CVB3 infection was not associated with the production of autoantibodies to PPP1R14C or FGL2. (**A**) Reactivity to PPP1R14C. Sera from infected animals positive (infected/PhIP-Seq^+^) or negative (infected/PhIP-Seq^−^) for PPP1R14C, along with sera from naïve mice were serially diluted and added in duplicates to plates coated with PPP1R14C. (**B**) Reactivity to FGL2. Sera obtained from infected animals along with sera for naïve mice were diluted (1:100) and added in duplicates to plates coated with FGL2. After washing and addition of HRP-conjugated anti-mouse total Ig and substrate, reactions were stopped to measure the OD values at 450 nm. Mean ± SEM values representing 4 to 6 samples in each group are shown. Two-way ANOVA with Tukey’s test was used to determine significance between groups. **** *p* ≤ 0.0001. Figure S4: Antibody reactivity to CVB3 VP1 from CVB3-infected animals revealed mainly IgG isotypes. Sera collected from CVB3-infected or naïve mice were serially diluted and added in duplicates to plates coated with CVB3 VP1. After adding HRP-conjugated anti-mouse IgM, IgG1, IgG2a, IgG2b, IgG3, and IgA as detection antibodies and substrate, reactions were stopped, and the OD values were measured at 450 nm. Mean ± SEM values representing a pool from 3 to 4 mice per sample in each group are shown (*n* = 3). Two-way ANOVA with Sidak’s test was used to determine significance between groups. * *p* ≤ 0.05 and **** *p* ≤ 0.0001. Figure S5: Reactivity of autoantibodies to COA4 in CVB3-infected mice did not reveal evidence of molecular mimicry. Sera obtained from CVB3-infected or naïve mice were diluted (1:100) and added in duplicates to plates coated with full-length CVB3 VP1 and COA4 proteins (positive control) and KLH and RNase 43-56 (negative control), in addition to mimicry peptide pairs (pair 1: CVB3 VP1 39–57 and COA4 1–19; pair 2: CVB3 VP1 295–310 and COA4 37–57). After adding HRP-conjugated anti-mouse total Ig and substrate, reactions were stopped, and OD values were measured at 450 nm. Mean ± SEM values representing 6 samples per group are shown. One-way ANOVA with Tukey’s test was used to determine significance between groups. **** *p* ≤ 0.0001. Table S1: Detection of antibodies to known autoantigens in the mouse peptide library.
