# Supplementary material for: PhIP-Seq Reveals Autoantibodies for Ubiquitously Expressed Antigens in Viral Myocarditis
Source: Biology (Basel). 2022 Jul 13;11(7):1055. doi: 10.3390/biology11071055 (PMC9312229; doi:10.3390/biology11071055)
Supplement: Supplementary file 1 [file biology-11-01055-s001.zip › Supplementary Table S1.pdf]

**Table S1. Detection of antibodies to known autoantigens in the mouse peptide library.**

| Gene | Autoantigen | Naïve (n=10) | Infected (n=10) |
|---|---|---|---|
| *Myh6* | Myosin-6 | 1 | 1 |
| *Myh7* | Myosin-7 | 1 | 4 |
| *Tnni3* | Troponin I, cardiac muscle (Fragment) | 5 | 5 |
| *Chrm2* | Muscarinic acetylcholine receptor M2 | 0 | 0 |
| *Slc25a4* | ADP/ATP translocase 1 | 0 | 0 |
| *Adrb1* | Beta-1 adrenergic receptor | 0 | 0 |
| *Bckdk* | [3-methyl-2-oxobutanoate dehydrogenase kinase, mitochondrial | 0 | 0 |
| *Agtr1a* | Type-1 angiotensin II receptor | 0 | 1 |
| *Actc1* | Actin, alpha cardiac muscle 1 (Fragment) | 0 | 0 |
| *Lama4* | Laminin subunit alpha-4 | 0 | 1 |
