# Supplementary figures and images for: PhIP-Seq Reveals Autoantibodies for Ubiquitously Expressed Antigens in Viral Myocarditis
Source: Biology (Basel). 2022 Jul 13;11(7):1055. doi: 10.3390/biology11071055 (PMC9312229; doi:10.3390/biology11071055)

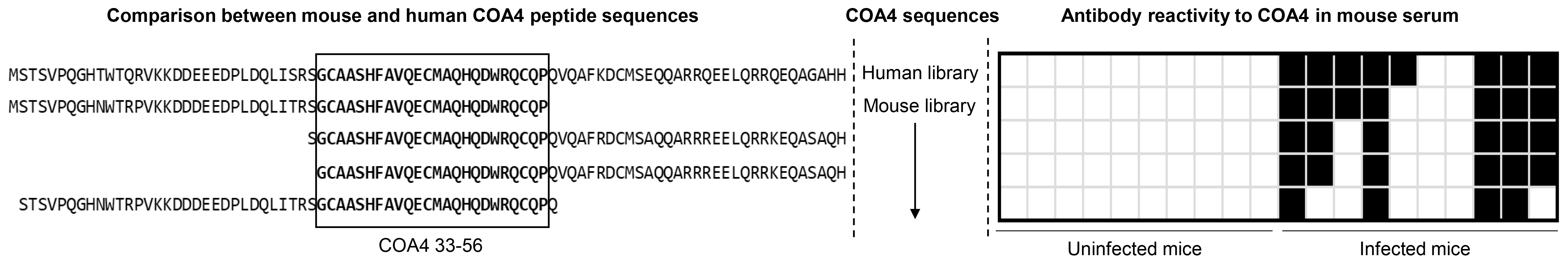

Supplement: Supplementary file 1 [file biology-11-01055-s001.zip › Supplementary Figure S1 MR.tif]

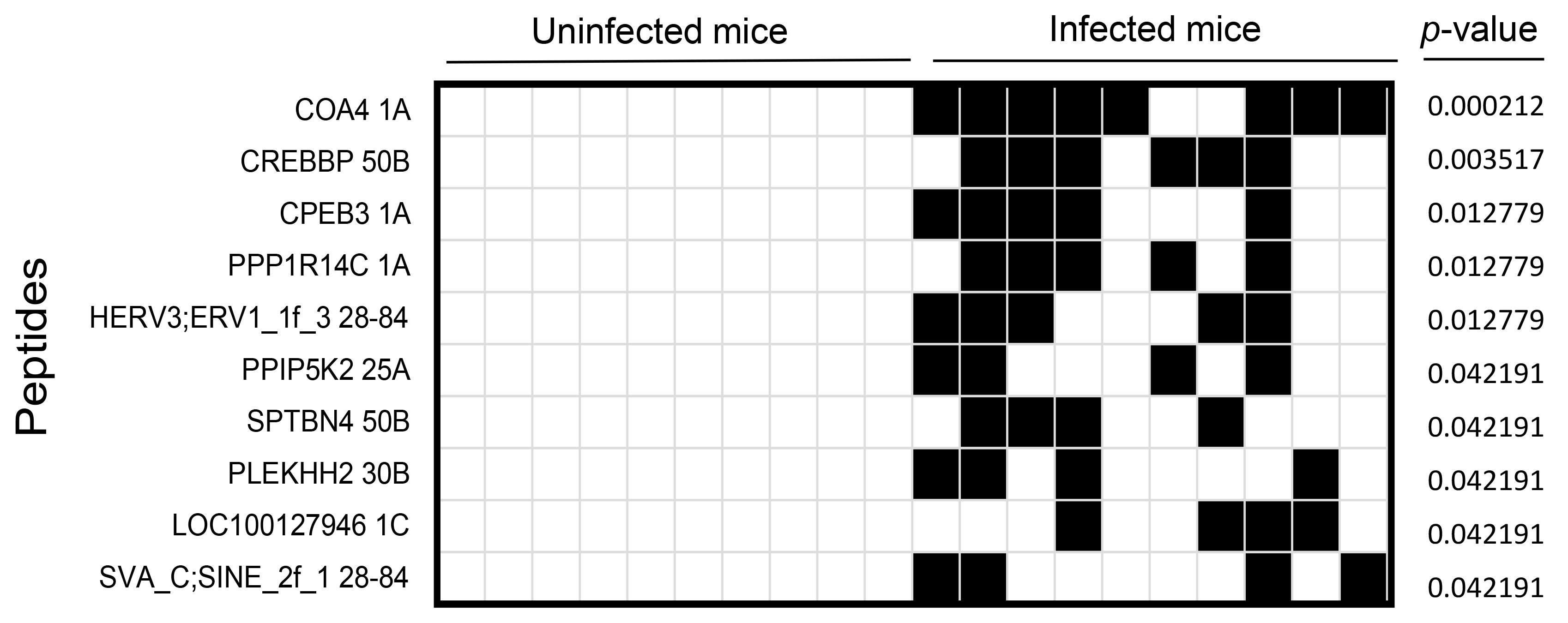

Supplement: Supplementary file 1 [file biology-11-01055-s001.zip › Supplementary Figure S2 MR.tif]

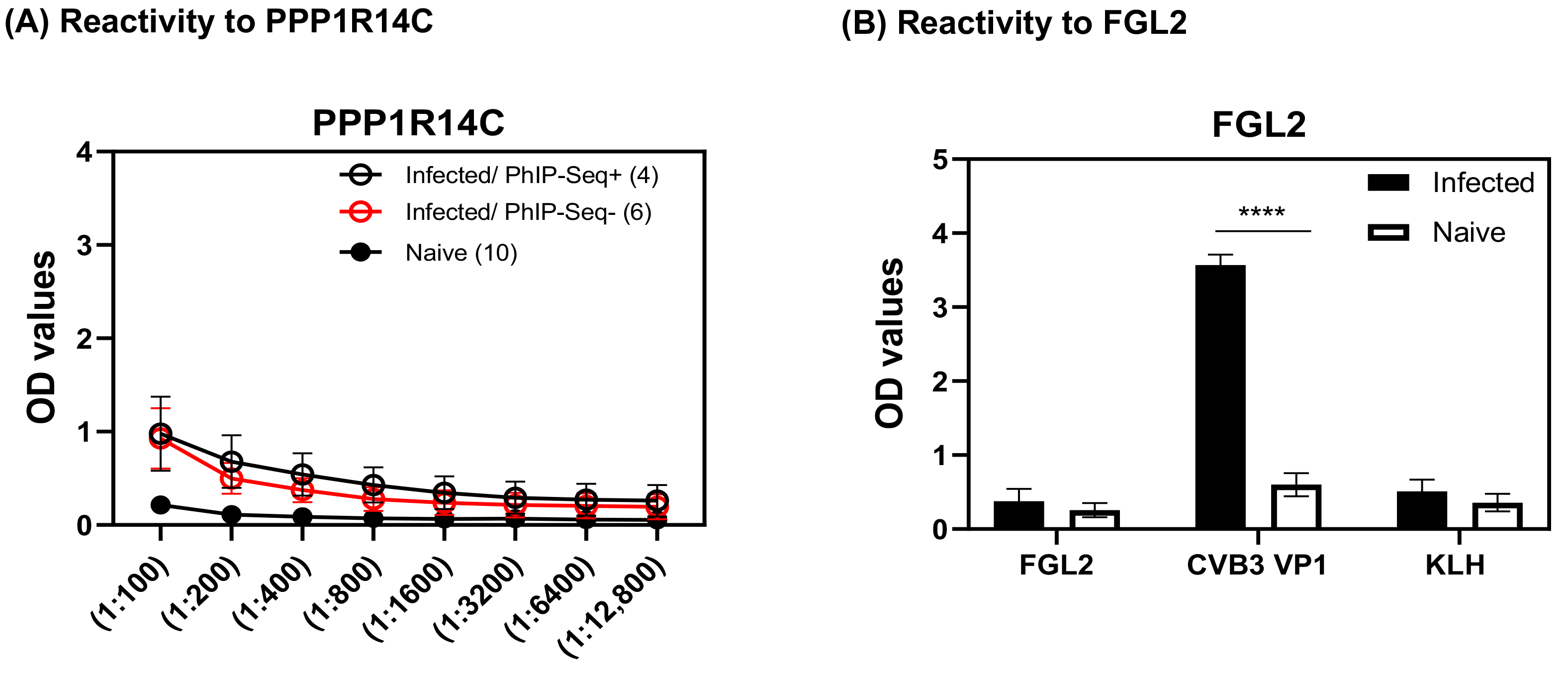

Supplement: Supplementary file 1 [file biology-11-01055-s001.zip › Supplementary Figure S3 MR.tif]

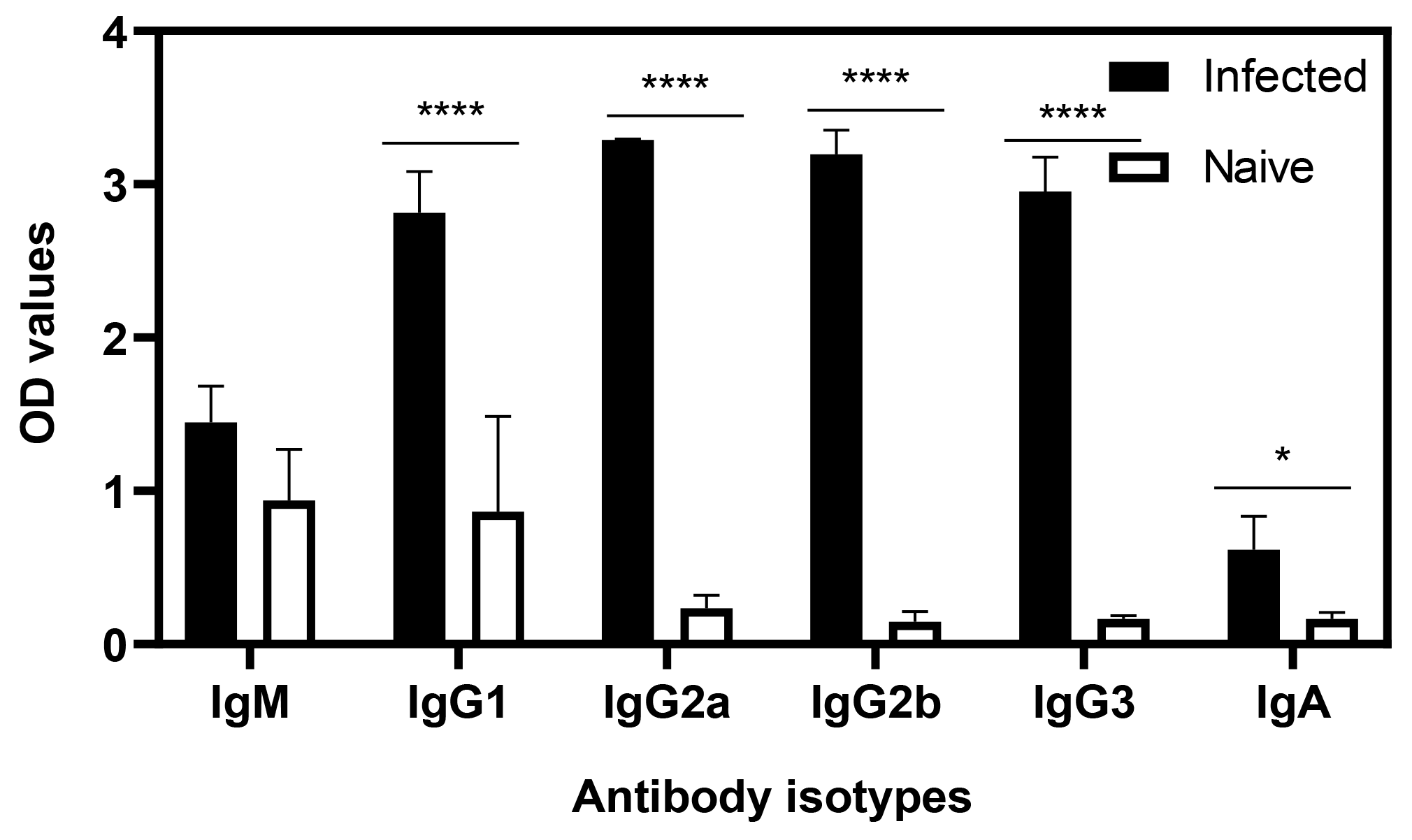

Supplement: Supplementary file 1 [file biology-11-01055-s001.zip › Supplementary Figure S4 MR.tif]

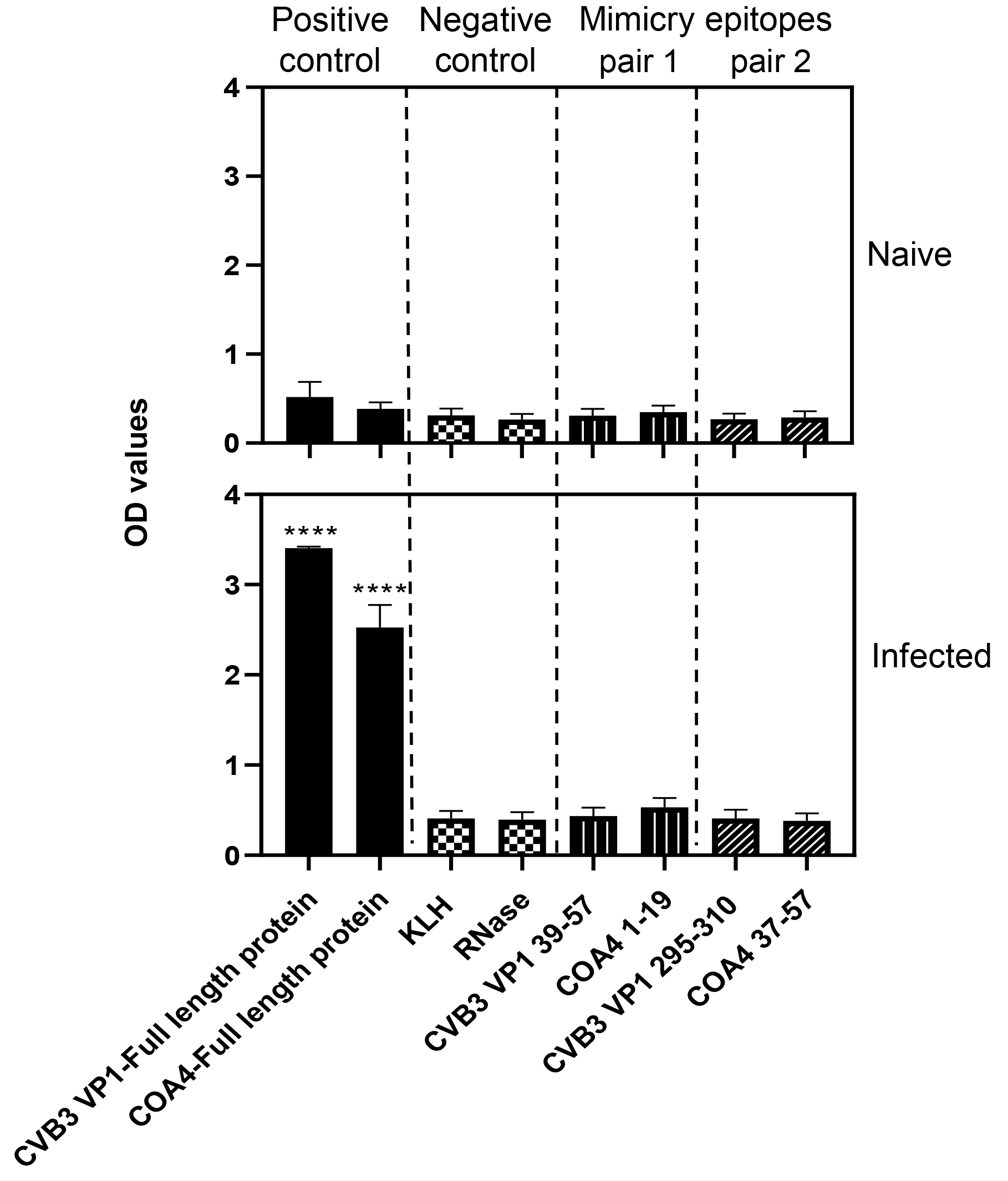

Supplement: Supplementary file 1 [file biology-11-01055-s001.zip › Supplementary Figure S5 MR.tif]
